# Changes in glycemic control and skeletal muscle mass indices after dapagliflozin treatment in individuals with type 1 diabetes mellitus

**DOI:** 10.1111/jdi.14054

**Published:** 2023-07-09

**Authors:** Yuta Yoshimura, Yoshitaka Hashimoto, Hiroshi Okada, Maya Takegami, Hanako Nakajima, Tomoki Miyoshi, Takashi Yoshimura, Masahiro Yamazaki, Masahide Hamaguchi, Michiaki Fukui

**Affiliations:** ^1^ Department of Endocrinology and Metabolism Kyoto Prefectural University of Medicine, Graduate School of Medical Science Kyoto Japan; ^2^ Department of Metabolism and Immunology Saiseikai Suita Hospital Suita Japan; ^3^ Department of Diabetes and Endocrinology Matsushita Memorial Hospital Moriguchi Japan; ^4^ Department of Metabolism and Immunology Japanese Red Cross Kyoto Daini Hospital Kyoto Japan

**Keywords:** Sarcopenia, Skeletal muscle mass, Sodium–glucose cotransporter 2 inhibitor

## Abstract

**Aims/Introduction:**

Dapagliflozin is used for individuals with type 1 diabetes, although the effect of this medication on skeletal muscle mass is not well established. In addition, there are few studies examining the effects of good glycemic control on skeletal muscle mass in type 1 diabetes patients. We investigated changes in glycemic control and skeletal muscle mass with dapagliflozin in individuals with type 1 diabetes, and the association between these changes.

**Materials and Methods:**

This was a post‐hoc analysis of a multicenter, open‐label, non‐randomized, prospective, interventional study in individuals with type 1 diabetes. The participants received dapagliflozin at 5 mg/day for 4 weeks, and were reviewed before and after treatment. Weight‐ and height‐corrected appendicular skeletal muscle mass (ASM) were calculated as indices of skeletal muscle mass using bioelectrical impedance analysis.

**Results:**

A total of 36 individuals were included in the analysis. After the 4 weeks of dapagliflozin treatment, ASM/height^2^ decreased in the body mass index <23 group (*P* = 0.004). ASM / weight decreased in all men aged >60 years. The change in ASM / weight (%) was negatively correlated with the change in glycated hemoglobin (%;*P* = 0.023). The change in ASM / height^2^ (kg/m^2^) was also positively correlated with the change in time within the glucose range of 70–180 mg/dL (*P* = 0.036).

**Conclusion:**

Dapagliflozin treatment of individuals with type 1 diabetes, particularly non‐obese individuals and older men, might result in loss of skeletal muscle mass. However, good glycemic control during treatment might prevent the onset and progression of sarcopenia.

## Graphical abstract

Individuals (*n* = 36) with type 1 diabetes received a dapagliflozin dose of 5 mg/day, and were reviewed before and after treatment. Based on weight‐ and height‐corrected appendicular skeletal muscle mass, we found evidence that non‐obese people and older men might be susceptible to loss of muscle mass, although good glycemic control during the treatment period should minimize the occurrence and progression of sarcopenia.

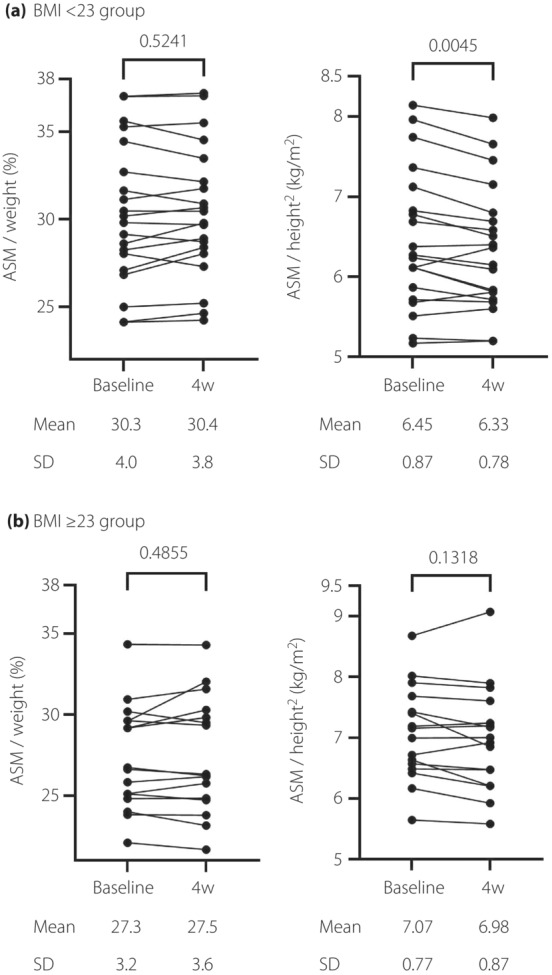

## INTRODUCTION

Sarcopenia is the age‐related loss of skeletal muscle mass and strength, which is associated with impaired mobility, falls and fractures, a decline in activities of daily living, and increased risk of death^1^. Skeletal muscle also plays a major role in glucose metabolism, and sarcopenia is a recognized complication of diabetes^2^. Individuals with diabetes mellitus, regardless of disease type, are at high risk for the incidence and progression of sarcopenia^3^, ^4^, ^5^, ^6^. With the recent increase in the number of individuals with diabetes globally, there is a need for effective measures to prevent functional impairment and physical disability in these individuals.

The relationship between glycemic control and sarcopenia in individuals with diabetes has been previously noted. For example, hyperglycemia is associated with muscle weakness^7^, and treatment with oral hypoglycemic agents might improve muscle function and inhibit muscle mass loss^8^. In a longitudinal observational study examining the relationship between glycemic control and sarcopenia in type 2 diabetes patients, height‐adjusted appendicular skeletal muscle mass (ASM) and walking speed increased in the group whose glycated hemoglobin (HbA1c) level decreased by ≥1% over 1 year, independent of exercise habits^9^. In contrast, few studies have examined whether good glycemic control has a positive effect on sarcopenia in individuals with type 1 diabetes.

Currently, dapagliflozin, a sodium–glucose cotransporter 2 inhibitor (SGLT2i), is recognized as a novel treatment for both type 1 and type 2 diabetes, and is used as an additional therapy to insulin to improve glycemic control and prevent complications of diabetes. However, muscle mass loss in individuals with type 2 diabetes using SGLT2i has also been observed^10^, ^11^. Although SGLT2i medication and accompanying insulin reduction might cause muscle mass loss, there is, in comparison, limited evidence of skeletal muscle mass changes during SGLT2i medication in type 1 diabetes.

In the present study, our group investigated the changes in glycemic control and body composition in individuals with type 1 diabetes after the use of dapagliflozin. We also examined the relationship between glycemic control changes and skeletal muscle changes.

## MATERIALS AND METHODS

### Study design and individuals

We undertook a post‐hoc analysis of a multicenter, open‐label, non‐randomized, prospective, interventional study, which is named as the RISING‐STAR study^12^, ^13^. The study design and patient population have been described previously^12^, ^13^. Briefly, 60 participants with type 1 diabetes between the ages of 20 and 80 years, HbA1c <10.5%, and body mass index (BMI) >18.5 kg/m^2^ were enrolled. The individuals were started on a dapagliflozin dose of 5 mg/day administered for 4 weeks. Each received a 10% reduction in insulin dose before dapagliflozin administration, and was able to titrate both basal and bolus insulin according to the given algorithm throughout the study. All participants were instructed to carry out home self‐ketone measurements every morning, and were instructed to stop taking dapagliflozin and consult the investigators if the measurements exceeded 600 μm/L^12^. The primary measured outcome was the daily frequency of hypoglycemia during the intervention period. Severe hypoglycemia is defined as the presence of hypoglycemic symptoms that cannot be managed by self alone and a venous plasma glucose level <60 mg/dL or capillary whole blood <50 mg/dL at onset, detection or visit^14^. Excluding the four individuals who withdrew from the study, of the remaining 56 participants, 36 whose body composition could be measured before and after the intervention were included in the post‐hoc analysis. The 36 participants were all able to take dapagliflozin continuously for the 4‐week trial period. The RISING‐STAR study was registered with the Japan Registry of Clinical Trials (jRCTs051190114), and approved by the ethics committees of the Kyoto Prefectural University of Medicine (CRB5200001) and complied with the Declaration of Helsinki. All participants gave written informed consent.

### Measurement

Blood tests were carried out at the baseline and at week 4 (referring to the last observation day, 4 weeks after the intervention) to measure blood glucose and HbA1c. BMI and body composition were measured using Inbody 720 (InBody Co., Ltd., Seoul, Korea), a non‐invasive body composition analyzer, at the baseline and at week 4. Other than body fat percentage (total fat mass / body weight [%]) and ASM (kg), weight‐adjusted ASM (ASM / bodyweight [%]) and height‐adjusted ASM (ASM / height^2^ [kg/m^2^]) were calculated as indicators of skeletal muscle mass. The study participants measured their blood glucose levels continuously at home using the Freestyle Libre system (Abbott Japan Co. Ltd.). The frequency of time (%) spent above range (TAR; blood glucose level >180 mg/dL), below range (TBR; <70 mg/dL) and within range (TIR; 70–180 mg/dL) was evaluated for 4 weeks before and after the intervention. The average insulin dose for the 3 days before the baseline and week 4 was used to determine the daily insulin use at each time point.

### Statistical analysis

Baseline characteristics were summarized as frequency (%) for categorical variables and as mean ± standard deviation (SD) for continuous variables. The associations between baseline skeletal muscle mass and other measured variables were examined using unpaired *t*‐tests or Pearson's correlation coefficients. Changes in glycemic control and body composition from the baseline to week 4 were examined using paired *t*‐tests. Participants were stratified by age, and body composition changes between groups were examined using the Mann–Whitney test. Correlations between variables were examined by calculating Pearson's correlation coefficient or Spearman's rank correlation coefficients. We also carried out the stratified analysis based on BMI ≥23.0 kg/m^2^, which has been proposed as a cut‐off for the diagnosis of overweight in Asian people^15^. This definition of overweight has often been used in the Japanese population^16^, ^17^. All statistical analyses were carried out using JMP software version 14.0.0 (SAS Institute Inc., Cary, NC, USA).

## RESULTS

The changes of skeletal muscle mass for 4 weeks of dapagliflozin combination therapy were evaluated in 36 participants with type 1 diabetes (12 men and 24 women; Table 1). Examining the association between baseline skeletal muscle mass and characteristics, ASM / weight (mean 32.4 ± 3.1%) and ASM / height^2^ (7.58 ± 0.68 kg/m^2^) of men were higher than those of women (27.3 ± 3.2% and 6.3 ± 0.61 kg/m^2^; *P* < 0.0001 and *P* < 0.0001, respectively). The ASM / weight (26.2 ± 3.2%) and ASM / height^2^ (6.43 ± 0.46 kg/m^2^) of the group with neuropathy were lower than those of the group without neuropathy (29.3 ± 3.8%, 6.75 ± 0.94 kg/m^2^; *P* = 0.07 and *P* = 0.44, respectively). Baseline BMI was negatively correlated with ASM /˜ weight (*r* = −0.51, *P* = 0.002), but was positively correlated with ASM / height^2^ (*r* = 0.46, *P* = 0.005). We found no apparent correlation between age, HbA1c, TAR, TBR, TIR, weight‐corrected total insulin use and skeletal muscle mass (ASM / weight: *r* = 0.06, *P* = 0.72; *r* = −0.29, *P* = 0.09; *r* = −0.18, *P* = 0.27; *r* = 0.05, *P* = 0.76; *r* = 0.17, *P* = 0.31; *r* = −0.22, *P* = 0.21, respectively. ASM / height^2^: *r* = 0.08, *P* = 0.65; *r* = −0.15, *P* = 0.39; *r* = 0.13, *P* = 0.45; *r* = 0.01, *P* = 0.97; *r* = −0.14, *P* = 0.41; *r* = −0.06, *P* = 0.72, respectively).

**Table 1:** Summary characteristics of the study cohort

	Mean (SD), or %
No. participants	36 (12 men, 24 women)
Age (years)	55.2 (12.7)
Duration of diabetes (years)	16.6 (12.3)
Bodyweight (kg)	61.4 (9.7)
BMI (kg/m^2^)	23.4 (3.2)
Plasma glucose (mg/dL)	186.0 (69.9)
HbA1c (%)	7.81 (0.89)
TAR (%)	29.4 (16.8)
TBR (%)	9.3 (9.9)
TIR (%)	61.3 (15.0)
C‐peptide (ng/mL)	0.31 (1.21)
Total insulin (unit/day)	36.8 (11.8)
Total insulin (unit/day/kg)	0.59 (0.15)
Basal insulin (unit/day)	13.1 (5.8)
Basal insulin (unit/day/kg)	0.21 (0.09)
Bolus insulin (unit/day)	23.7 (9.0)
Bolus insulin (unit/day/kg)	0.38 (0.12)
Total fat mass (kg)	17.8 (6.3)
Body fat percentage (%)	28.7 (7.5)
ASM (kg)	17.8 (3.9)
ASM / weight (%)	29.0 (4.0)
ASM / height^2^ (kg/m^2^)	6.72 (0.87)
Coexisting diseases	
Hypertension (%)	22.2
Dyslipidemia (%)	38.9
Diabetic complications	
Nephropathy (%)	22.2
Retinopathy (%)	8.8
Neuropathy (%)	18.2
Macrovascular complications (%)	2.8
Insulin pump user (%)	11.1

Data are expressed as frequencies (percentages) for categorical variables or mean (standard deviation) for continuous variables. ASM, Appendicular skeletal muscle mass; BMI, body mass index; HbA1c, glycated hemoglobin; SD, standard deviation; TAR, time spent above range; TBR, time spent below range; TIR, time spent within range.

Table 2 summarizes the changes in glycemic control parameters and body composition after the intervention. HbA1c decreased from 7.81 ± 0.89 to 7.48 ± 0.77% (*P* < 0.0001) during the study. TAR decreased from 29.4 ± 16.8 to 17.3 ± 11.9%, TBR increased from 9.3 ± 9.9 to 13.0 ± 14.0%, resulting in an increase in TIR from 61.3 ± 15.0 to 69.8 ± 12.3% (*P* < 0.0001, *P* = 0.001, and *P* = 0.0002, respectively). Neither severe hypoglycemia nor diabetic ketoacidosis was observed in these 36 participants. The doses of total insulin and basal insulin decreased from 0.59 ± 0.15 to 0.57 ± 0.17 unit/day/kg (*P* = 0.04) and from 0.21 ± 0.09 to 0.19 ± 0.08 unit/day/kg (*P* < 0.0001), respectively. Bodyweight, BMI and body fat percentage decreased from 61.4 ± 9.7 to 60.2 ± 9.5 kg (*P* = 0.0001), 23.4 ± 3.2 to 22.9 ± 3.1 kg/m^2^ (*P* = 0.0001) and 28.7 ± 7.5 to 28.3 ± 7.3% (*P* = 0.09), respectively. ASM / height^2^ decreased from 6.72 ± 0.87 to 6.62 ± 0.87 kg/m^2^ (*P* = 0.002), whereas ASM/weight did not change (29.0 ± 4.0 to 29.1 ± 3.9%, *P* = 0.33).

**Table 2:** Changes (paired *t*‐test) in glycemic control parameters and body composition from the baseline to week 4

	Baseline	Week 4	*P*
Plasma glucose (mg/dL)	186 (69.9)	167.7 (53.8)	0.16
Change from baseline		−18.3 (76.7)	
HbA1c (%)	7.81 (0.89)	7.48 (0.77)	<0.0001
Change from baseline		−0.33 (0.36)	
TAR (%)	29.4 (16.8)	17.3 (11.9)	<0.0001
Change from baseline		−11.6 (11.6)	
TBR (%)	9.3 (9.9)	13 (14.0)	0.001
Change from baseline		3.7 (5.9)	
TIR (%)	61.3 (15.0)	69.8 (12.3)	0.0002
Change from baseline		7.9 (11.0)	
Total insulin (unit/day)	36.8 (11.8)	34.7 (13.5)	0.014
Rate of change from baseline (%)		−6.9 (13.0)	
Total insulin (unit/day/kg)	0.59 (0.15)	0.57 (0.17)	0.04
Change from baseline		−0.02 (0.08)	
Basal insulin (unit/day)	13.1 (5.8)	11.6 (5.2)	<0.0001
Rate of change from baseline (%)		−11.0 (11.5)	
Basal insulin (unit/day/kg)	0.21 (0.09)	0.19 (0.08)	<0.0001
Change from baseline		−0.02 (0.02)	
Bolus insulin (unit/day)	23.7 (9.0)	23.0 (10.5)	0.43
Rate of change from baseline (%)		−3.4 (19.9)	
Bolus insulin (unit/day/kg)	0.38 (0.12)	0.37 (0.14)	0.64
Change from baseline		−0.01 (0.08)	
Bodyweight (kg)	61.4 (9.7)	60.2 (9.5)	0.0001
Change from baseline		−1.22 (1.04)	
Rate of change from baseline (%)		−1.96 (1.52)	
BMI (kg/m^2^)	23.4 (3.2)	22.9 (3.1)	0.0001
Change from baseline		−0.46 (0.39)	
Rate of change from baseline (%)		−1.96 (1.52)	
Body fat percentage (%)	28.7 (7.5)	28.3 (7.3)	0.09
Change from baseline		−0.46 (1.59)	
ASM (kg)	17.8 (3.9)	17.6 (3.8)	0.003
Change from baseline		−0.28 (0.51)	
Rate of change from baseline (%)		−1.5 (2.7)	
ASM / bodyweight (%)	29.0 (4.0)	29.1 (3.9)	0.33
Change from baseline		0.12 (0.74)	
ASM / height^2^ (kg/m^2^)	6.72 (0.87)	6.62 (0.87)	0.002
Change from baseline		−0.11 (0.19)	
Rate of change from baseline (%)		−1.53 (2.73)	
No. events during observation			
Sever hypoglycemia	0		
Diabetic ketoasidosis	0		

Data are expressed as mean (standard deviation). ASM, appendicular skeletal muscle mass; BMI, body mass index; HbA1c, glycated hemoglobin; TAR, time spent above range; TBR, time spent below range; TIR, time spent within range.

**Figure 1:**
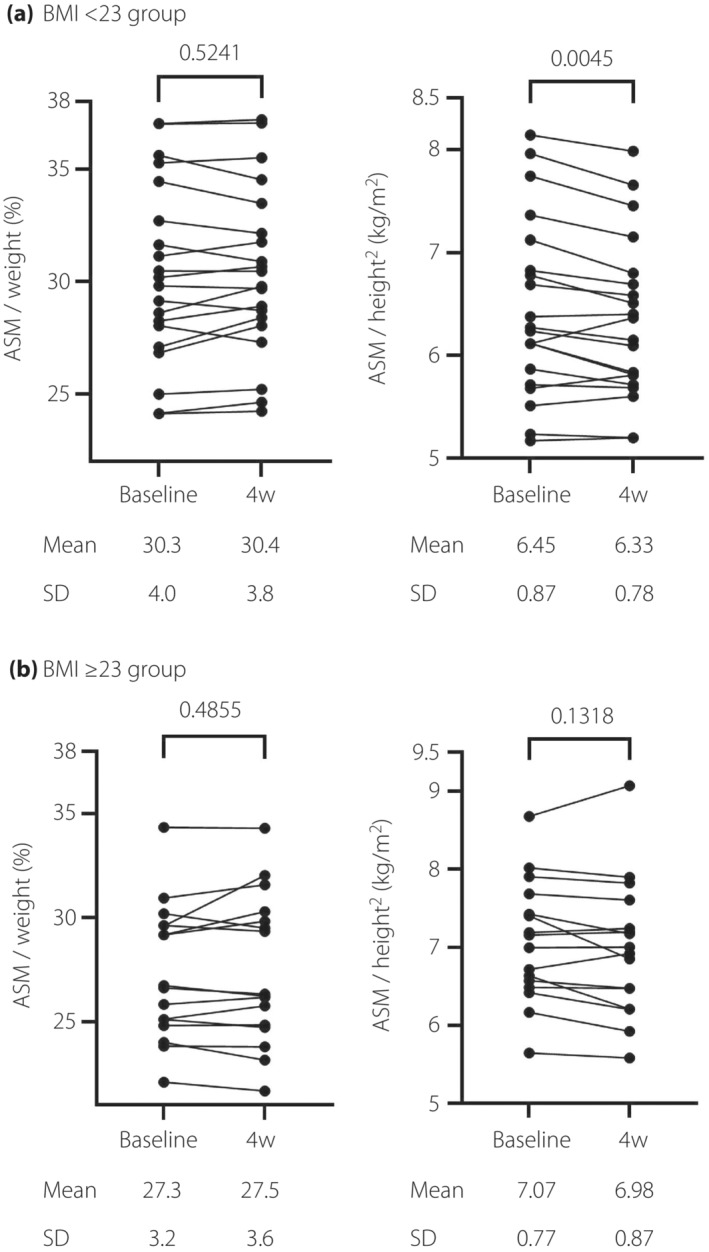
Comparison (paired *t*‐test) of skeletal muscle mass at the baseline and week 4 (4w). (a) Changes in the body mass index (BMI) <23 group (*n* = 20). (b) Changes in the BMI ≥23 group (*n* = 16). ASM, appendicular skeletal muscle mass.

When stratified by baseline BMI, ASM / height^2^ decreased in the BMI <23 group (*n* = 20) from 6.45 ± 0.87 to 6.33 ± 0.78 kg/m^2^ (*P* = 0.005; Figure 1a). In the BMI ≥23 group (*n* = 16), ASM / height^2^ also decreased, from 7.07 ± 0.77 to 6.98 ± 0.87 kg/m^2^ (*P* = 0.13; Figure 1b). ASM / weight did not change in either group (BMI <23 group: 30.3 ± 4.0 to 30.4 ± 3.8%, *P* = 0.52; BMI ≥23 group: 27.3 ± 3.2 to 27.5 ± 3.6%, *P* = 0.49).

**Figure 2:**
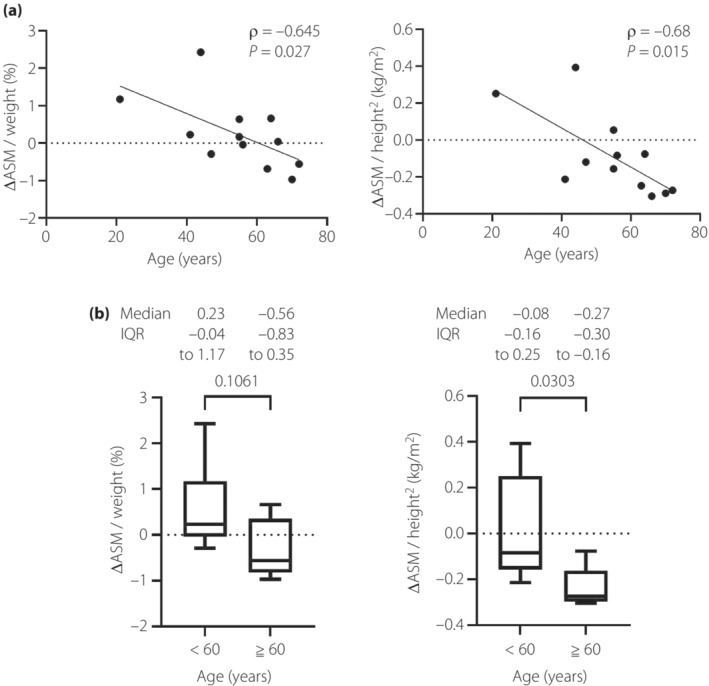
Relationships between age and skeletal muscle mass change in men (*n* = 12). (a) Correlation between age and changes in skeletal muscle mass using Spearman's rank correlation coefficient. (b) Difference in skeletal muscle mass changes between the age <60 years group (*n* = 7) and the age ≥60 years group (*n* = 5) using the Mann–Whitney test. The line in the middle of the box indicates the median value; the box extends from the 25th to the 75th percentiles. *ρ*, Spearman's rank correlation coefficient. ASM, appendicular skeletal muscle mass; IQR, interquartile range.

When the participants were grouped by sex, age and skeletal muscle mass change were negatively correlated in men (*n* = 12; ΔASM / weight [%]: *ρ* = −0.645, *P* = 0.027; ΔASM / height^2^ (kg/m^2^): *ρ* = −0.68, *P* = 0.015; Figure 2a), but were not correlated in women (*n* = 24). When the male subjects were stratified by age, all five men aged ≥60 years showed a decrease in ASM/height^2^ compared with only four of the seven men aged <60 years (Figure 2b). ΔASM / weight in men aged ≥60 years was negative compared with men aged <60 years (median −0.56, interquartile range −0.83 to 0.35% vs 0.23, −0.04 to 1.17%; *P* = 0.11), as was ΔASM / height^2^ (median −0.27, IQR −0.30 to −0.16 kg/m^2^ vs –0.08, −0.16 to 0.25 kg/m^2^; *P* = 0.03).

Finally, the change in ASM / weight (%) was negatively correlated with changes in TAR (*r* = −0.37, *P* = 0.026) and HbA1c (*r* = −0.38, *P* = 0.023; Table 3). The change in ASM / height^2^ (kg/m^2^) was also negatively correlated with the change in TAR (*r* = −0.37, *P* = 0.027) and HbA1c (*r* = −0.52, *P* = 0.001), but positively correlated with the change in TIR (*r* = 0.36, *P* = 0.036). Both BMI and body fat percentage changes were negatively correlated with TBR change (*r* = −0.35, *P* = 0.04; *r* = −0.36, *P* = 0.035, respectively). Insulin dose change (units/day/kg) was not associated with changes in body composition.

**Table 3:** Correlation (Pearson's correlation coefficient) between changes in glycemic control parameters and changes in body composition

	ΔTAR (%)		ΔTBR (%)		ΔTIR (%)		ΔHbA1c (%)	
	*r*	*P*	*r*	*P*	*r*	*P*	*r*	*P*
Bodyweight (kg)								
Change from baseline	0.028	0.87	−0.28	0.10	0.12	0.49	−0.29	0.084
Rate of change from baseline (%)	0.016	0.93	−0.35	0.04	0.17	0.33	−0.28	0.098
BMI (kg/m^2^)								
Change from baseline	0.030	0.87	−0.27	0.12	0.11	0.52	−0.27	0.11
Rate of change from baseline (%)	0.016	0.93	−0.35	0.04	0.17	0.33	−0.28	0.098
Body fat percentage (%)								
Change from baseline	0.36	0.033	−0.36	0.035	−0.18	0.28	0.21	0.22
ASM / bodyweight (%)								
Change from baseline	−0.37	0.026	0.26	0.12	0.25	0.14	−0.38	0.023
ASM / height^2^ (kg/m^2^)								
Change from baseline	−0.37	0.027	0.07	0.69	0.36	0.036	−0.52	0.001
Rate of change from baseline (%)	−0.34	0.044	0.06	0.73	0.33	0.054	−0.52	0.001

	ΔTotal Is (unit/day/kg)		ΔBasal Is (unit/day/kg)		ΔBolus Is (unit/day/kg)	
	*r*	*P*	*r*	*P*	*r*	*P*
Bodyweight (kg)						
Change from baseline	0.00	0.99	0.19	0.27	−0.06	0.72
Rate of change from baseline (%)	0.04	0.82	0.24	0.15	−0.04	0.82
BMI (kg/m^2^)						
Change from baseline	−0.02	0.86	0.19	0.26	−0.09	0.60
Rate of change from baseline (%)	0.04	0.82	0.24	0.15	−0.04	0.82
Body fat percentage (%)						
Change from baseline	−0.08	0.66	0.13	0.45	−0.12	0.50
ASM / bodyweight (%)						
Change from baseline	0.18	0.30	−0.13	0.45	0.21	0.21
ASM / height^2^ (kg/m^2^)						
Change from baseline	0.20	0.23	−0.02	0.92	0.20	0.23
Rate of change from baseline (%)	0.20	0.25	0.00	0.99	0.19	0.26

ASM, appendicular skeletal muscle mass; BMI, body mass index; HbA1c, glycated hemoglobin; Is, insulin; *r*, Pearson's correlation coefficient; Tar, time spent above range; TBR, time spent below range; TIR, time spent within range.

## DISCUSSION

ASM / height^2^ has been used as an index to evaluate skeletal muscle mass in sarcopenia^18^; however, this index is highly correlated with BMI, the standard for obesity. The index mainly identifies thinner people as sarcopenic, and might underestimate sarcopenia in overweight people^19^. Therefore, many studies use ASM / weight to assess relative skeletal muscle mass. In the present results, ASM / height^2^ was positively correlated with BMI, as previously reported, thus we used both ASM / weight and ASM / height^2^ as skeletal muscle mass indices.

We found that ASM / height^2^, but not ASM / weight decreased after starting dapagliflozin medication. The present findings also suggest that the addition of dapagliflozin to individuals with type 1 diabetes, but without obesity, might reduce skeletal muscle mass. In contrast, we could not clarify how dapagliflozin affects muscle mass in individuals with a BMI above a certain level. It has been reported that plasma glucagon concentrations and hepatic glycogenesis increase with energy loss due to urinary glucose excretion in individuals on SGLT2i^20^. Substrates for glycogenesis include glycerol from adipose tissue and glycogenic amino acids from skeletal muscle. In non‐obese individuals, body fat as an energy source might be limited and, consequently, skeletal muscle might be prone to catabolism.

The present study shows that SGLT2i administration might have an unfavorable effect on skeletal muscle mass in older men with type 1 diabetes. It is widely known that the absolute amount of skeletal muscle mass is higher in men than in women^21^, and this is thought to be due to the influence of sex hormones. Testosterone is related to changes in skeletal muscle mass in men, and plays an important role in muscle hypertrophy and muscle loss prevention by regulating insulin‐like growth factor‐1, and activating protein synthesis and satellite cells^22^, ^23^, ^24^. It has also been reported that age‐related muscle mass loss is greater in men than in women^21^, ^25^. Long‐term administration of SGLT2i to older men with type 1 diabetes might result in synergistic loss of muscle mass due to aging during treatment, although the development and progression of sarcopenia during SGLT2i administration requires future research. It is known that the prevalence of sarcopenia is higher in older individuals with type 2 diabetes than in younger people^26^, but such effects are not clear in individuals with type 1 diabetes. In the present study, we found no association between age and skeletal muscle mass in either men or women.

Good glycemic control in individuals with type 1 diabetes has been established to prevent the development of microvascular diseases^27^. In contrast, we have not found any studies that have longitudinally examined the relationship between glycemic control and muscle mass index in type 1 diabetes patients. In the current study, we found that changes in ASM / weight (%) were negatively correlated with changes in TAR and HbA1c, and changes in ASM / height^2^ (kg/m^2^) were further positively correlated with changes in TIR. These results suggest that good glycemic control in type 1 diabetes patients might contribute to the prevention and further progression of sarcopenia. No association was found between sarcopenia and glucose control in a cross‐sectional study of individuals with diabetes^28^, and the present results also show no clear association between baseline glycemic control and muscle mass index.

Skeletal muscle function is impaired in individuals with type 1 diabetes^4^, and factors linking diabetes and sarcopenia include decreased insulin signals, chronic inflammation, mitochondrial dysfunction and peripheral neuropathy^29^, ^30^, ^31^, ^32^. Furthermore, insulin use contributes to the preservation of muscle mass in type 1 diabetes patients and is thought to prevent sarcopenia^33^. Changes in total insulin (unit/day/kg), basal insulin (unit/day/kg) and bolus insulin (unit/day/kg) were also not associated with changes in skeletal muscle mass. As the number of individuals with neuropathy was only six, a detailed study of the relationship between the presence of neuropathy and skeletal muscle mass is a future priority.

Decreases in bodyweight, BMI and body fat, and increases in TBR, were observed with the start of SGLT2i treatment, and these changes were correlated. The decrease in body fat might represent energy production from adipose tissue due to hypoglycemia^34^. Importantly, when rapid weight loss is observed after SGLT2i medication administration, as well as a positive effect related to improvements in insulin resistance, attention must also be paid to an increased frequency of hypoglycemia.

The key limitations of the present study were that it was a post‐hoc analysis based on short‐term results with no control participants. Furthermore, we did not collect indicators of muscle function, such as grip strength and walking speed. In contrast, our analysis provides valuable evidence of changes in the two skeletal muscle mass parameters. The study sample size of 36 participants was not enough to generalize the findings from this study. In contrast, it is necessary to accumulate the effects of dapagliflozin in type 1 diabetes patients. Indeed, Martínez‐Montoro *et al*.^35^ reported the benefit of dapagliflozin combination therapy in a retrospective analysis of 38 participants with type 1 diabetes treated with dapagliflozin at two centers. Both nutritional statuses, including protein intake and the amount of exercise, were not considered. Participants receive no exercise or dietary advice/instruction during the intervention period. Future research should consider such variables to further enhance our understanding of dapagliflozin treatment effects on type 1 diabetes individuals. Overall, the present results show that the use of dapagliflozin might result in the loss of skeletal muscle mass in non‐obese individuals and in older men with type 1 diabetes. In contrast, good glycemic control during treatment might prevent the onset and progression of sarcopenia.

## DISCLOSURE

MH received grants from AstraZeneca K.K., Ono Pharma Co. Ltd. and Kowa Pharma Co. Ltd., and received personal fees from AstraZeneca K.K., Ono Pharma Co. Ltd., Eli Lilly, Japan, Sumitomo Dainippon Pharma Co., Ltd., Daiichi Sankyo Co. Ltd., Mitsubishi Tanabe Pharma Corp., Sanofi K.K., K.K. and Kowa Pharma Co. Ltd. outside of the submitted work. MY received grants from AstraZeneca K.K., and Ono Pharma Co. Ltd., and received personal fees from MSD K.K., Sumitomo Dainippon Pharma Co., Ltd., Kowa Company, Limited, AstraZeneca PLC, Takeda Pharmaceutical Company Limited, Kyowa Hakko Kirin Co., Ltd., Daiichi Sankyo Co., Ltd, Kowa Pharmaceutical Co., Ltd. and Ono Pharmaceutical Co., Ltd. outside the submitted work. YH received personal fees from Novo Nordisk Pharma Ltd., Sanofi K.K., Sumitomo Dainippon Pharma Co., Ltd., Nippon Boehringer Ingelheim Co., Mitsubishi Tanabe Pharma Corp., Kowa Company, Ltd., Taisho Pharma Co., Eli Lilly Japan K.K. and Daiichi Sankyo Co. MF received grants from Ono Pharma Co. Ltd., Oishi Kenko inc., Yamada Bee Farm, Nippon Boehringer Ingelheim Co. Ltd., Kissei Pharma Co. Ltd., Mitsubishi Tanabe Pharma Corp., Daiichi Sankyo Co. Ltd., Sanofi K.K., Takeda Pharma Co. Ltd., Astellas Pharma Inc., MSD K.K., Kyowa Kirin Co., Ltd., Sumitomo Dainippon Pharma Co., Ltd., Kowa Pharma Co. Ltd., Novo Nordisk Pharma Ltd., Sanwa Kagagu Kenkyusho CO., Ltd., Eli Lilly, Japan, K.K., Taisho Pharma Co., Ltd., Terumo Corp., Tejin Pharma Ltd., Nippon Chemiphar Co., Ltd., Abbott Japan Co. Ltd., Johnson & Johnson K.K. Medical Co. and Terumo Corporation, and received personal fees from Nippon Boehringer Ingelheim Co., Ltd., Kissei Pharma Co., Ltd., Mitsubishi Tanabe Pharma Corp., Daiichi Sankyo Co. Ltd., Sanofi K.K., Takeda Pharma Co. Ltd., Astellas Pharma Inc., MSD K.K., Kyowa Kirin Co. Ltd., Sumitomo Dainippon Pharma Co. Ltd., Kowa Pharma Co. Ltd., Novo Nordisk Pharma Ltd., Ono Pharma Co. Ltd., Sanwa Kagaku Kenkyusho Co. Ltd., Eli Lilly Japan K.K., Taisho Pharma Co., Ltd., Bayer Yakuhin, Ltd., AstraZeneca K.K., Mochida Pharma Co. Ltd., Abbott Japan Co. Ltd., Teijin Pharma Ltd., Arkray Inc., Medtronic Japan Co. Ltd., Nipro Corp. and Terumo Corporation outside the submitted work.

Approval of the research protocol: The ethics committees of the Kyoto Prefectural University of Medicine approved the RISING‐STAR study on 12 December 2019 (CRB 5200001). The study was carried out under the principles of the Declaration of Helsinki.

Informed consent: Informed consent was obtained in the form of opt‐in.

Approval date of Registry and the Registration No. of the study: The RISING‐STAR study was registered with the Japan Registry of Clinical Trials on 2 March 2020 (jRCTs051190114).

Animal studies: N/A.

## DATA AVAILABLITY STATEMENT

The data used in this study and the datasets analyzed are available from the corresponding author upon request.
